# Alterations in the Three Components of Selfhood in Persons with Post-Traumatic Stress Disorder Symptoms: A Pilot qEEG Neuroimaging Study

**DOI:** 10.2174/1874440001812010042

**Published:** 2018-04-30

**Authors:** Andrew A. Fingelkurts, Alexander A. Fingelkurts

**Affiliations:** *BM-Science – Brain and Mind Technologies Research Centre, Espoo, Finland*

**Keywords:** Self-referential brain network, Default-mode network, DMN, Subjective sense of self, First-person perspective, Electroencephalogram, EEG, Alpha rhythm, Operational synchrony, Functional connectivity, PTSD, Traumatic events

## Abstract

**Background and Objective:**

Understanding how trauma impacts the self-structure of individuals suffering from the Post-Traumatic Stress Disorder (PTSD) symptoms is a complex matter and despite several attempts to explain the relationship between trauma and the “Self”, this issue still lacks clarity. Therefore, adopting a new theoretical perspective may help understand PTSD deeper and to shed light on the underlying psychophysiological mechanisms.

**Methods:**

In this study, we employed the “three-dimensional construct model of the experiential selfhood” where three major components of selfhood (phenomenal first-person agency, embodiment, and reflection/narration) are related to three Operational Modules (OMs) of the self-referential brain network. These modules can be reliably estimated through operational synchrony analysis of the Electroencephalogram (EEG). Six individuals with PTSD symptoms and twenty-nine sex-, age- and demographic- (race, education, marital status) matched healthy controls underwent resting state EEG signal acquisition with the following estimation of the synchrony strength within every OM.

**Results:**

Our results indicate that subjects with PTSD symptoms had significantly stronger EEG operational synchrony within anterior and right posterior OMs as well as significantly weaker EEG operational synchrony within left posterior OM compared to healthy controls. Moreover, increased the functional integrity of the anterior OM was positively associated with hyperactivity symptoms, reduced synchrony of the left posterior OM was associated with greater avoidance, and increased right posterior OM integrity was positively correlated with intrusion and mood symptoms.

**Conclusion:**

The results are interpreted in light of the triad model of selfhood and its theoretical and clinical implications (including a new treatment approach) are discussed.

## 1 INTRODUCTION

Post-Traumatic Stress Disorder (PTSD) develops in some people after exposure to a traumatizing event (a scary, shocking, or dangerous event) that involves either an actual or threatened death, a serious injury or threat to the physical integrity of oneself or others, with concurrent feeling of helplessness, fear, or horror by the witnessing/involved person. The fifth edition of Diagnostic and Statistical Manual of Mental Disorders (DSM-5) classifies the condition as “a trauma- and stress-related disorder” [1]. Persons, who acquire PTSD after a traumatic/stressful event, invariably experience a well-defined set of symptoms that clustered in four categories under DSM-5: hyperarousal, persistent re-experiencing of the trauma, avoidance of trauma-related stimuli, as well as negative alterations in cognition and mood [1]. It is a persistent and debilitating condition [2], and although some therapeutic interventions can be effective, many PTSD sufferers do not achieve sufficient improvement or long-term remission [3-5] Hence, it is suggested that new and theory-based treatment options are needed [6]; and for that a new theoretical perspective should be taken on this disorder that would allow to understand deeper the psychophysiological mechanisms of PTSD [6-9] and help devise novel prevention and treatment options [6].

A fear conditioning mediated by the neurocircuits that involve amygdala dominates current major theoretical models of PTSD [10, 11]. This conceptualization has been largely based on MRI and fMRI studies, in which it has been shown that low volumes of hippocampus and ventromedial prefrontal cortex (vmPFC), as well as altered activity in the amygdala, vmPFC, hippocampus and insular cortex are responsible for the diminished capacity of the brain to inhibit fear and failure to adaptively maintain extinction of conditioned emotional responses (for the review see Ref. [11]). While such a view is relevant, there is an abundant body of evidence that point to the possibility that *PTSD is fundamentally a disorder of the self* [12-15]. Indeed, it has been repeatedly observed that the traumatic event is neither symbolized nor properly coded/conceptualized and thus lacks the linguistic/contextual/narrative components of the autobiographical self [15-17]. At the same time, the traumatic experience is encoded bodily *via* the sensory/motor/somatic/emotional states that tend to persistently reoccur as intrusive memories, which are experienced in “nowness” of the embodied self [15, 18-21]. Additionally, the traumatic experience results in hypervigilance (or hyperarousal) and increased self-focus which is often responsible for anger, aggression [22, 23] and/or self-destructive behavior [6, 24]. Thus it has been proposed that in subjects suffering from PTSD, the traumatic experience is akin to “black hole” that engulfs into itself every aspect of the self, resulting in substantial distortion to the overall sense of selfhood [15].

Therefore, a “self” or more broadly “selfhood” could be considered a useful theoretical framework to study PTSD aiming to deepen our understanding of it. Specifically, it would be important to use the neurophysiological model of selfhood that would help to elucidate the neurophysiological and pathophysiological mechanisms behind such a disorder. This perspective is consistent with a recent trend in NIMH framework for mental health research that focuses on identifying risks and neurophysiologic markers of specific symptomatic/functional domains that cut across traditional diagnostic categories (the Research Domain Criteria (RDoC) approach [25, 26]).

Recently, such neurophysiological model has been proposed under the name of “*three-dimensional construct model of the experiential selfhood”* [27, 28]. It is based on a broad analysis of current empirical findings about the complex role of self-referential brain network (also referred to as default mode network) [29-32], and on the functional-topographical specialization of three major subnets (or Operational Modules, OM) of this network during normal/healthy states [32-35], altered states of self-consciousness [27, 28] and during pathological states when self-consciousness is either seriously affected such as in depression [36] or is minimal or even lost completely as in disorders of consciousness [37-39]. This model also considers and is congruent with the multi-faceted nature of self-awareness [40-43] where three major components of selfhood (phenomenal first-person agency, embodiment and reflection/narration) could be distinguished [44-46]. As shown previously [27, 28, 32, 36-39], the three OMs responsible for the aforementioned triad of self-related qualities can be reliably estimated through operational synchrony analysis of the electroencephalogram (EEG) signal [47, 48]. Thus, this model offers a useful and practical “tool” that enables studying the triad of separate, though closely related qualities characterizing self-referential processing, and which together form a unified sense of self [32].

According to the “triad” model of selfhood [27, 28], the *frontal OM* of self-referential brain network is responsible for the phenomenal first-person perspective and the sense of agency in the sense that it is ‘I’ who is undergoing an experience in its implicit first-person mode of givenness [44, 45], – it could be subsumed as a “witnessing observer” or simply the “Self” in a narrow sense. The *right posterior OM* of the self-referential brain network is involved in the experience of self as a localized embodied entity (by means of interoceptive and exteroceptive bodily sensory processing), related emotion states, and autobiographical memories as multisensory percepts [27, 28], – it could be shortly designated as a “representational-emotional agency” or simply “Me”. The *left posterior OM* of the self-referential brain network is responsible for the experience of thinking about and reflecting upon oneself, including narration and inner speech (and autobiographical narrative), as well as linguistic reinterpretation of short-term memory events related to self [27, 28], – it could be shortly termed as “reflective agency” or simply “I”.

The internal integrity of all three OMs and related to them, three aspects/qualities of selfhood get altered in a predictable way when the sense of complex self is altered as for example in meditation [27, 28], depression [36], or when it is very minimal or completely lost due to a brain damage [37, 38]. Moreover, a six-year longitudinal analysis of (self-)consciousness recovery after severe brain injury has confirmed that dynamics of functional integrity of the self-referential brain network’s OMs are indeed coupled with different aspects of complex selfhood, such as first-person agency (or “Self”), representational-emotional agency (or “Me”), and reflective agency (or “I”) and are corroborated by the findings from the clinical examinations and observations [39].

Thus, considering this neurophysiological three-dimensional model of complex selfhood, in the present study, we sought to investigate potential disturbances in the integrity of three OM of the self-referential brain network during the resting state in individuals with PTSD symptoms. Capitalizing on the previously discussed observations that PTSD sufferers have increased hypervigilance/hyperarousal and enhanced sensory/motor/somatic/emotional states coupled with simultaneous lack of narration and linguistic/contextual aspects about traumatic experience, we *hypothesized* that they would exhibit increased integrity in both the frontal and right posterior OMs alongside diminished integrity of the left posterior OM. To date, no studies have examined whether there is the differential integrity of the submodules of the self-referential brain network in the PTSD and relation of such submodules to altered different aspects of selfhood in PTSD.

To do so, for the purpose of this study, we applied the neurophysiological triad model of selfhood to a nonclinical population with *subthreshold levels* of PTSD [49, 50]. This was done intentionally to reduce potential confounding from other comorbid psychiatric conditions (like depression, anxiety), usage of psychotropic medications and treatment history. Additionally, this strategy is justified by the fact that individuals with subthreshold PTSD differ in the same way as PTSD patients from individuals who have been traumatized but did not develop symptoms of PTSD [51, 52]. Furthermore, both subthreshold PTSD and clinical PTSD sufferers showed the same distinctive pattern of EEG activity when compared to traumatized individuals without PTSD symptoms and nontraumatized controls [53].

## 2 METHODS

### 2.1 Subjects

For the purpose of this study archived EEG, demographic and medical data of subjects who had experienced a traumatic event were extracted for retrospective analysis from the data-registry of BM-Science (*N* = 245 on the day of study onset). These subjects (*N* = 8) have been invited for the interview and were administered Posttraumatic Stress Disorder Symptom Scale-Interview for DSM-5 (PSSI-5)^1^ [55]. Participants were eligible to be included in the current study if they met subthreshold PTSD criteria [52]. These criteria require at least one symptom in each of the symptom clusters that last at least one month [52]. Based on the PSSI-5 screening, 6 subjects (1 male, 5 females; mean age 40 ± 13 years) met the criteria for subthreshold PTSD (*sub-PTSD*) and were included in this study. The mean PTSD score was 29.33 ± 19. Analysis of medical data records revealed that besides having sub-PTSD symptoms, subjects were in otherwise good physical health and did not use psychoactive medications. Exclusion criteria included a clinical diagnosis of depression, anxiety, history of schizophrenia, alcohol or drug dependence within 5 years preceding the EEG registration, neurological disorders or having sustained brain concussion, and usage of psychoactive medications. The demographic data and traumatic events of the 6 subjects with sub-PTSD symptoms are presented in Table **1**.

For the healthy control group (*CONTR*) data from 29 sex- and age- and demographic- (race, education, marital status) matched nonsmoking healthy subjects (5 males, 24 females, mean age 41 ± 9 years) were used. The data of control subjects were included in the study if such subjects did not experience any traumatic event and did not have a history of neurological or psychiatric pathology, as well never used psychotropic medication. The PSSI-5 was not applied for control subjects because they were trauma naive.

Both groups (sub-PTSD and CONTR) were screened using the Beck Anxiety Inventory (BAI) [56], Beck Depression Inventory (BDI) [57], and Big Five Inventory (BFI) [58] to assess neuroticism as a personality trait of 

We choose PSSI instead of Clinician-Administered PTSD Scale (CAPS), because CAPS psychometric properties in nonveteran populations are not well studied and CAPS has quite long assessment time; at the same time, it has been shown that PSSI is reliable and valid in civilian trauma sufferers, correlates strongly with CAPS and requires significantly less assessment time when compared with CAPS [54].

negative emotionality, including anxiety, depression, and anger [59]. Table (**2**) provides mean values of these scales as well as some demographic characteristics (sex, age, race, education, marital status) for the sub-PTSD and CONTR groups.

This study was carried out in accordance with the Code of Ethics of the World Medical Association (Declaration of Helsinki) and standards established by the BM-Science – Brain and Mind Technologies Research Centre Review Board. Prior to EEG scanning, the experimental procedures were explained and participants signed an informed consent form. The use of the data for scientific studies was authorized by written informed consent of subjects and approval by the Review Board of BM-Science – Brain and Mind Technologies Research Centre.

### 2.2 EEG Registration and Pre-Processing

The EEG was recorded using a 21-channel EEG data acquisition system (Mitsar, St. Petersburg, Russian Federation) from 19 electrodes positioned on the head according to the International 10–20 system (*i.e.* O1, O2, P3, P4, Pz, C3, C4, Cz, T3, T4, T5, T6, Fz, F3, F4, F7, F8, Fp1, Fp2) during waking resting state with eyes closed. Additionally, the following recording parameters were enforced: linked earlobes as a reference electrode; 0.5–30 Hz bandpass; 50 Hz notch filter ON; 250 Hz sampling rate; electrooculogram (0.5–70 Hz bandpass); 6-min eyes closed. The impedance was below 5–10 kΩ.

EEG recordings were done late in the morning when the participants were asked to relax and engage in no specific mental activity. The presence of an adequate EEG-signal was determined by visual inspection of the raw signal. Artefacts due to eyes opening, eye movement, significant muscle activity, and movements on EEG channels, as well as drowsy episodes (indexed by slowing of background frequencies by ≥1 Hz, vertex sharp waves and slow eye movements) were corrected or eliminated by (a) using spatial filtration technique based on zeroing the activation curves of individual Independent Component Analysis (ICA) components that correspond to these artefacts [60], as well as (b) excluding epochs with excessive amplitude of EEG (≥ 70 μV) and excessive fast (20-35 Hz, ≥ 35 μV) and slow (0-1 Hz, ≥ 50 μV) frequency activity.

For every registration, a full artifact-free EEG stream was fragmented into consecutive 1-minute epochs, which were bandpass-filtered (sixth order Butterworth filter) in the alpha (7–13 Hz) frequency band. Phase shifts were eliminated by forward and backward filtering. The alpha frequency band was chosen because: (1) it has been repeatedly demonstrated that it is the alpha rhythm (among other EEG frequency bands) that has a significant positive correlation with self-referential brain network [61-65]; (2) alpha oscillations dominate the EEG of humans in the absence of external stimuli when mind-wandering and spontaneous thoughts are most pronounced [66-70]; (3) it has been shown that exactly EEG alpha band operational connectivity within three modules of self-referential network correlates significantly with variation of self-consciousness during brain pathology [37-39] and with selfhood alterations during meditation [27, 28].

### 2.3 Estimation of Self-Referential Network OMs and Their Strength

As in the previous EEG studies [27, 28, 36-39], a constellation of nine operationally synchronized cortical areas was used to estimate the operational synchrony strength within the three OMs^2^ (*frontal OM*: F3-Fz-F4; *left posterior OM*: T5-P3-O1; and *right posterior OM*: T6-P4-O2).

To estimate the operational synchrony strength within every OM, several stages of data processing were required. The details of these procedures can be found elsewhere [47, 48]. A brief overview of the main steps is provided here. As the *first step*, each local EEG signal was reduced to a temporal sequence of nearly stationary (quasi-stationary) segments of varying duration. To uncover these quasi-stationary segments from the complex nonstationary structure of local EEG signals, an adaptive segmentation procedure was used [47, 48]. The aim of such segmentation is to divide each local EEG signal into naturally existing quasi-stationary segments by estimating the intrinsic boundaries among segments – Rapid Transitional Periods (RTPs). An RTP is defined as an abrupt change in the analytical amplitude of the signal above a particular threshold, derived experimentally (and verified in modelling studies) based on statistical procedures [47, 48]. It has been proposed that each stationary (homogeneous) segment in the local EEG signal corresponds to a temporary stable microstate – an operation executed by a neuronal assembly [71]. The temporal coupling (synchronization) of such segments among several local EEG recordings then, reflects the synchronization of operations (*i.e.* operational synchrony), produced by different neuronal assemblies (located in different cortical regions) into integrated and unified patterns responsible for complex mental operations [71].

The *second step* of analysis signifies the estimation of operational synchrony. Measurement of operational synchrony estimates the statistical level of RTP temporal coupling between two or more local EEG recordings [47, 48]. The measurement tends towards zero if there is no synchronization between EEG segments derived from different EEG channels and has positive or negative values where such synchronization exists. Positive values (above upper stochastic threshold) indicate ‘active’ coupling of EEG segments (synchronization of EEG segments is observed significantly more often than expected by chance as a result of random shuffling of segments during a computer simulation), whereas negative values (below lower stochastic threshold) mark ‘active’ decoupling of segments (synchronization of EEG segments is observed significantly less than expected by chance as a result of random shuffling of segments during a computer simulation) [47, 48]. The strength of EEG operational synchrony is proportional to the actual (absolute) value of the measure: The higher this value, the greater the strength of the functional connectivity.

Using pair-wise analysis, operational synchrony was identified in several (more than two) channels – synchrocomplexes (SC); these define operational modules – OMs. The criterion for defining an OM is a sequence of the same synchrocomplexes (SC) during every 1-min epoch, whereas an SC is a set of EEG channels in which each channel forms a paired combination with valid values of synchrony with all other EEG channels in the same SC; meaning that all pairs of channels in an SC have to have statistically significant synchrony linking them together [47, 48].

It is frequently claimed that analysis performed at the sensor level is prone to volume conduction and it is the main obstacle in interpreting EEG data in terms of brain connectivity. The operational synchrony measure used in the current study has been specifically tested previously through modeling experiments to address this issue. Such studies have shown that the values of the operational synchrony are sensitive to the morpho-functional organization of the cortex rather than to the volume conduction, signal power, and/or reference electrode (for further details, we refer the reader to Ref. [ 47, 48]).

### 2.4 Statistics

The strength of functional connectivity within individual OMs was assessed using EEG operational synchrony analysis (see the previous subsection). The differences in strength of operational synchrony between sub-PTSD and CONTR groups were presented as a percent change from the control group and statistical significance was assessed

See Ref. [27] page 30 for the studies that clearly established the correlations between EEG activity in a given electrode position and its correspondent brain cortical area. These results have been verified through an EEG-MRI sensor system and an automated projection algorithm.

using Wilcoxon’s *t*-test. At first, all strength values of EEG operational synchrony were averaged within every OM for all 1-min EEGs per subject and then averaged for all subjects per group (sub-PTSD and CONTR). Differences between the demographic parameters and psychometric tests were assessed either by Wilcoxon’s *t*-test or by Chi-squared test. For correlation analysis of OMs integrity strength and PSSI-5 scores, the Spearman rank order correlation test was used.

## 3 RESULTS

### 3.1 Demographic Characteristics

A group comparison of the demographic and psychometric characteristics is shown in Table (**2**). The groups (sub-PTSD and CONTR) did not differ on important demographic variables: gender and race ratio, age (Wilcoxon's *t*-test, *p* = 0.79), education (χ^2^, *p* = 0.21) and marital status (χ^2^, *p* = 0.21). However, as expected, the mean anxiety, depression and neuroticism scores were significantly higher in the sub-PTSD group (Wilcoxon's *t*-test, *p* = 0.01, *p* = 0.005, and *p* = 0.0006 respectively) compared to CONTR group in which all these values were within the normal range.

### 3.2 Neurophysiological Findings

**Fig. (1):**
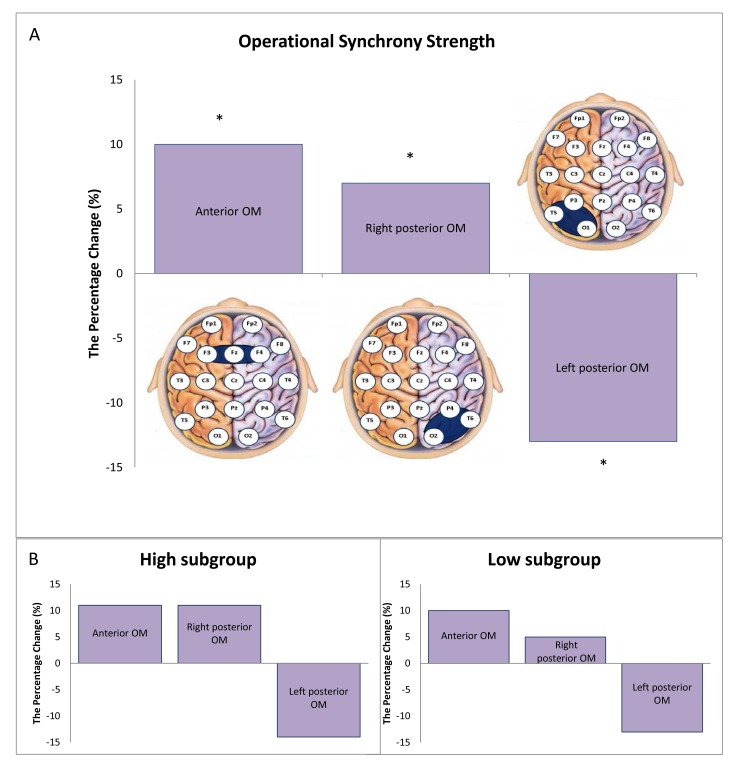
**Percent change from the control norm in the strength of operational synchrony within the three OMs of the brain self-referential network in the sub-PTSD group**. The *Y*-axis presents percent change. The *X*-axis represents the three OMs. The schematic cortex maps below and above the graphs indicate the positions of OMs (dark blue shapes). Abbreviations: OM: Operational Module; PTSD: Post-Traumatic Stress Disorder. * - *p* < 0.05.

In the sub-PTSD group, we observed a marked decrease in the strength of EEG operational synchrony within the left posterior OM with percent change -13% from the healthy control, while the strength of EEG operational synchrony within the right posterior OM and anterior OM increased with percent change +7% and +10%, respectively from the healthy control (Fig. **1A**). The difference in means was significant (Wilcoxon's *t*-test): *p* = 0.002 for anterior OM, *p* = 0.04 for right posterior OM and *p* = 0.03 for left posterior OM.

From the Table (**1**), it can be seen that three subjects in the sub-PTSD group (# 1, 3 and 6) have high scores in anxiety, depression, and neuroticism and three subjects (# 2, 4 and 5) have low scores. The score discrepancy may serve as a confounding covariate of the overall significant results; therefore, we conducted stratification analysis based on ‘high’ and ‘low’ anxiety, depression, and neuroticism scores (Fig. **1B**). The stratification analysis revealed the same change directions for both (‘high’ and ‘low’) subgroups as for the overall sub-PTSD group, thus the main result remained unchanged.

Correlations between the strength of EEG operational synchrony within every OM and PSSI-5 criteria are presented in Table (**3**). The increased anterior OM integrity was positively correlated with hyperactivity symptoms (criterion E according to DSM-5), decreased left posterior OM integrity was associated with greater avoidance (criterion C), and increased right posterior OM integrity was positively correlated with intrusion and mood symptoms (criteria B and D respectively). None of the three OMs correlated significantly with the overall PSSI-5 score.

## 4 DISCUSSION

To our knowledge, this is the first study examining functional integrity within three modules of the self-referential brain network related to three aspects of selfhood during the resting state in individuals with PTSD symptoms. In agreement with our initial hypothesis, we have found that persons with subthreshold PTSD symptoms exhibited a pattern with significantly stronger EEG operational synchrony within anterior OM and right posterior OM alongside significantly weaker EEG operational synchrony within left posterior OM when compared to healthy controls (Fig. **1A**).

Considering the functional roles of these three OMs in relation to different qualia of complex selfhood [27, 28], such as first-person agency or “Self” (anterior OM), representational-emotional agency or “Me” (right posterior OM), and reflective/narrative agency or “I” (left posterior OM), one can usefully interpret the main results of this study in relation to a specific set of PTSD symptoms. Increased synchrony/integrity of the right posterior OM coupled with decreased integrity of the left posterior OM (Fig. **1A**) may explain why the traumatic experience in PTSD sufferers consists of enhanced emotional, sensory and bodily states, with little verbal representation and lack of linguistic/contextual information [16]. Indeed, PTSD subjects report [15] that they have intrusive and unwanted memories (criterion B according to DSM-5) that are rich in multimodal (movie-like) mental images consisting of highly detailed bodily/sensory sensations of the traumatic event (criterion D according to DSM-5): fear, stress, frozenness, shivering, shaking, trembling, palpitations and sweating (see also [21]). Essentially, such traumatic memories are reduced to a specific fragmented moment, a moment without a story, without narration, and like a “black hole,” it engulfs the entire self [15]. This may explain why PTSD sufferers feel of being repeatedly sucked into a traumatic experience devoid of narrative framing.

Furthermore, since the left posterior OM is responsible for thinking about and reflecting upon oneself, including narration and inner speech [27, 28] – and this aspect of selfhood is a precondition for an experience of self-ownership of the body and thoughts [27], the decreased integrity of this OM may contribute to the impaired self-structure of PTSD sufferers who often feel unreal or experience thoughts and feelings as being outside the self [72] – avoidance (criterion C according to DSM-5). This finding clearly highlights the importance of narration for the integrity of self. Indeed, as pointed by van der Kolk and McFarlane [73] narration provides a sense of projected temporal continuation for the self that offers psychological promise to look beyond whatever difficulty one may have at the present moment, thus helping to build an ontological anchoring in a constantly changing world. When this process malfunctions, as in the traumatised individuals, they may find themselves fixating on trauma while at the same time trying to avoid any reminder of it [15, 73].

We have also found that in comparison to healthy controls, participants with PTSD symptoms showed increased synchrony/integrity in the anterior OM (Fig 1A). Since this OM is responsible for self-focus and self-perspective, it may contribute to the observed PTSD sufferers hypervigilance [6], associated increased anger and aggression [22], as well as self-destructive behaviours [24]. The set of these symptoms constitutes the criterion E according to DSM-5. Thus, we may hypothesise that very high integrity of the anterior OM may reflect the PTSD group individuals’ increased vigilance to their surroundings with a concurrent shift of their first-personal perspective from the current moment in time to the moment of the traumatic event. Perhaps, a sensory overload resulting from constant hypervigilance and profound emotional arousal further exacerbate alienation of the self [74].

Taking the findings of this study together, it seems unlikely that PTSD can be solely attributed to historically implicated altered connectivity in the fronto-limbic regions (for a similar view see [75, 76]). Our findings rather suggest that impaired functioning of three OMs of the self-referential brain network modulates three major aspects of selfhood, and thereby leads to the development of specific PTSD symptoms. Consistent with this proposal, we have found (see Table **3**), that increased functional integrity of the anterior OM was associated with hyperactivity symptoms (criterion E according to DSM-5), reduced left posterior OM integrity was associated with greater avoidance (criterion C), and increased right posterior OM integrity was associated with thought intrusion and mood symptoms (criteria B and D, respectively).

Depression, anxiety and neuroticism are very common in persons experiencing PTSD symptoms [77, 78], which raises the question as to whether the observed alterations in the integrity of OMs in the sub-PTSD group could be confounded by them. Indeed, half of the subjects participated in the current study had high scores in depression, anxiety and neuroticism (see Table **1**). To examine this possibility, the sub-PTSD group was stratified into ‘high’ and ‘low’ depression, anxiety and neuroticism score sub-groups. The main results of the study did not change as a result of this stratification (see Figs. **1A** and **B**), suggesting that increased synchrony within anterior and right posterior OMs and decreased synchrony within left posterior OM may be specific for the development of the PTSD pathophysiology. Thus, we may hypothesize that a failure in the regulation of the integrity in brain self-referential network modules, that are crucial for the healthy structure of the self, may play an etiological role in the development of PTSD.

## CONCLUSION

This study has both clinical and theory-based implications. The theory underlying this study emphasizes that the self constituted an integral, structural and organising core of an individual’s personality. Our findings both add to the growing evidence that conceptualizes the experiential selfhood in terms of self-referential processes [30], and also illuminate the link between such processes and their neurophysiological representation by means of a specially dedicated and operationally integrated brain network [29, 31, 79, 80]. Such self-referential brain network is quite heterogeneous – it consists of at least three distinct subnets or modules that serve separate but interacting qualities characterizing complex selfhood [27, 28]. As a whole, it organises overall human behaviour and mental life from a first-person perspective [32].

Clinically, this study suggests that in addition to the traditional model of altered fronto-limbic connectivity, the basis for PTSD may be malfunctioning in the integrity of three operational modules in the self-referential brain network resulting in an alteration in three aspects of the normal structure of self. Understanding how trauma impacts self-structure is a complex matter indeed, and despite several attempts to devise clinical models for the relation of trauma and self, these models lack sufficient explanatory power [81]. Our findings, with the help of the “three-dimensional construct model of the experiential selfhood” [27, 28], provide a novel potential aetiological account of PTSD symptoms grouped under DSM-5 in four clusters (hyperarousal, persistent re-experiencing of the trauma, avoidance of trauma-related stimuli, and negative alterations in cognition and mood), and also explain distinct contributions (related to three major aspects of selfhood: first-person agency, representational-emotional agency, and reflective/narrative agency) that every module of the self-referential brain network makes to PTSD. Our model also highlights the importance of simultaneous alterations in the integrity of all three modules that may drive the development PTSD symptoms.

If corroborated by further studies, these findings, guided by the triad model of selfhood, can enhance our understanding of the origins of PTSD beyond just simple danger exposure followed by fear circuity re-wiring and in such a way may help facilitate the development of new targeted/personalized therapies and prevention strategies. The benefits of such personalised approach are expected to result in better therapeutic outcomes, reduced suffering, increased safety and comfort for both affected individuals and persons who are in close relationships with them. For example, the following complex 3-part therapy utilising the results of this study could be proposed for future studies: (i) talking/writing about the traumatic event may help bring the left posterior OM back to normal baseline function, (ii) using medications that lessen the hyper-activity of anterior cortical areas (for example psilocybin, see [82]) may calm down overly active anterior OM, and (iii) using meditation (or other self-regulating techniques) may reduce right posterior OM synchrony (as was shown in [27, 28]). Future studies could assess the effects of interventions targeted at specific symptom clusters associated with specific neurophysiologic correlates across diagnostic boundaries (consistent with NIMH RDoC approach [25, 26]). Further, if the findings of the present study are replicated in larger samples of patients with diagnosed PTSD, and the correlations between specific PTSD symptom clusters and specific OMs remain, there could be a potential role for this EEG-based tool as a non-invasive biomarker for subcomponents of PTSD, *i.e.*, disturbances in self-referential functions.

### Limitations

The study has several limitations. First, our study sample size was small, predominantly female and consisted of participants who had non-combat-related traumas. It is unknown whether the findings of the present study would generalize to males and combat-related traumas. The results of this study should be validated in a prospective study with a larger and more representative samples size. Second, this study focused on persons with subthreshold levels of PTSD. Whilst it was documented that individuals with subthreshold PTSD differ (also neurophysiologically) in the same way as PTSD patients from traumatized individuals without PTSD symptoms and nontraumatized controls [51-53], future studies should validate the present findings on patients that meet clinical PTSD criteria. Third, in this study, the subjects with subthreshold PTSD symptoms were compared with a healthy control population, but not (additionally) with trauma-exposed individuals without PTSD symptoms. Thus, future studies should consider this additional control. Despite these limitations, the major strengths of the current study included thorough clinical assessment, close matching of participants with PTSD symptoms to a neurologically- and psychiatrically-healthy comparison group, and state-of-the-art EEG analysis based on the neurophysiological triad model of selfhood [27, 28].

## Figures and Tables

**Table 1:** Demographic data of sub-PTSD group.

Subject	Age	Gender	BAI	BDI	BFI	PSSI-5	Trauma	Time Since Trauma
								
1	33	M	35	35	4.6	43	Father's disease, fear of death	108 months
2	41	F	5	6	1.6	10	Fall from height	360 months
3	18	F	16	16	4.5	52	Averse reaction to lumbar puncture, fear of death	3 months
4	44	F	8	4	2.3	16	Work accident	25 months
5	50	F	2	6	2.5	11	Life-threatening illness, fear of death	480 months
6	52	F	40	12	3.6	44	Physical abuse by aggressive father	250 months

Sub-PTSD: subthreshold post-traumatic stress disorder group. BAI: Beck Anxiety Inventory, BDI: Beck Depression Inventory, BFI: Big Five Inventory/neuroticism, PSSI-5: Posttraumatic Stress Disorder Symptom Scale Interview for DSM-5.

**Table 2:** Study groups characteristics.

Characteristic	sub-PTSD	CONTR	Statistics	*p*-Value
Gender (% of males)	17	17	–	–
Age (years) ± SD	40 ± 13	41 ± 9	W=-0.26	0.79
Race (% of Caucasian)	100	100	–	–
Education (% ≥15 years)	67	75	X²=1.55	0.21
Marital status (% of married)	67	75	X²=1.55	0.21
BAI ± SD	18 ± 16	4 ± 3	W=2.86	0.01
BDI ± SD	13 ± 12	2 ± 1.8	W=3.18	0.005
BFI ± SD	3.2 ± 1.2	1.7 ± 0.3	W=4.23	0.0006

**Table 3:** Spearman rank order correlations.

–	Criterion E	Criterion B	Criterion D	Criterion C
–	*Hyperactivity*	*Intrusion*	*Mood*	*Avoidance*
Anterior OM	**0.76**	0.43	0.66	0.29
Left posterior OM	-0.08	-0.45	-0.34	**-0.76**
Right posterior OM	0.63	**0.84**	**0.77**	0.62

Marked correlations are significant at *p*<0.05.
